# Can the Combined Use of Two Screening Instruments Improve the Predictive Power of Dependency in (Instrumental) Activities of Daily Living, Mortality and Hospitalization in Old Age?

**DOI:** 10.14283/jfa.2019.17

**Published:** 2019-06-12

**Authors:** Linda P.M. Op het Veld, E. van Rossum, G.I.J.M. Kempen, A.J.H.M. Beurskens, K.J. Hajema, H.C.W. de Vet

**Affiliations:** 1 Centre of Research Autonomy and Participation for Persons with a Chronic Illness, Faculty of Health, Zuyd University of Applied Sciences, P.O. Box 550, 6400 AN, Heerlen, the Netherlands; 2 CAPHRI, Care and Public Health Research Institute, Department of Health Services Research, Maastricht University, P.O. Box 616, 6200 MD, Maastricht, the Netherlands; 3 CAPHRI, Care and Public Health Research Institute, Department of Family Practice, Maastricht University, P.O. Box 616, 6200 MD, Maastricht, the Netherlands; 4 Community Health Service South Limburg, Academic Collaborative Centres Public Health (ACC), P.O. Box 33, 6400 AA, Heerlen, the Netherlands; 5 Department of Epidemiology and Biostatistics, Amsterdam Public Health research institute, Amsterdam University Medical Centers, location VU University, De Boelelaan 1089A, 1081 HV, Amsterdam, the Netherlands

**Keywords:** Frail older people, frailty (instruments), screening, sensitivity and specificity, combined use

## Abstract

**Background:**

Due to differences in the definition of frailty, many different screening instruments have been developed. However, the predictive validity of these instruments among community-dwelling older people remains uncertain.

**Objective:**

To investigate whether combined (i.e. sequential or parallel) use of available frailty instruments improves the predictive power of dependency in (instrumental) activities of daily living ((I)ADL), mortality and hospitalization.

**Design, setting and participants:**

A prospective cohort study with two-year follow-up was conducted among pre-frail and frail community-dwelling older people in the Netherlands.

**Measurements:**

Four combinations of two highly specific frailty instruments (Frailty Phenotype, Frailty Index) and two highly sensitive instruments (Tilburg Frailty Indicator, Groningen Frailty Indicator) were investigated. We calculated sensitivity, specificity, positive predictive value (PPV), and negative predictive value (NPV) for all single instruments as well as for the four combinations, sequential and parallel.

**Results:**

2,420 individuals participated (mean age 76.3 ± 6.6 years, 60.5% female) in our study. Sequential use increased the levels of specificity, as expected, whereas the PPV hardly increased. Parallel use increased the levels of sensitivity, although the NPV hardly increased.

**Conclusions:**

Applying two frailty instruments sequential or parallel might not be a solution for achieving better predictions of frailty in community-dwelling older people. Our results show that the combination of different screening instruments does not improve predictive validity. However, as this is one of the first studies to investigate the combined use of screening instruments, we recommend further exploration of other combinations of instruments among other study populations.

## Introduction

Life expectancy is increasing in most Western countries, resulting in larger populations of older and frail older people (1). Although the debate concerning the conceptualization of frailty is ongoing, there is consensus that being frail increases the risk of adverse outcomes, such as mortality, hospitalization and functional decline (2). The variety in definitions has led to the development and use of many different instruments to identify frail community-dwelling older people; however, the predictive validity of these instruments is generally limited (3).

In a recent study, Op het Veld and colleagues investigated the ability of various indices to predict mortality, hospitalization and dependency in (instrumental) activities of daily living ((I)ADL), namely: the Frailty Phenotype (FP), the Groningen Frailty Indicator (GFI), the Tilburg Frailty Indicator (TFI) and the Frailty Index (FI) (4). All frailty instruments performed poorly in predicting mortality, hospitalization and (I)ADL dependency (area under the receiver operating characteristic curve [AUC] 0.62–0.65, 0.59–0.63 and 0.60–0.64, respectively). Several other studies have demonstrated somewhat more positive outcomes. A study of Gobbens and colleagues showed one of the highest AUCs: 0.80–0.83 for the TFI in predicting (I)ADL disability over a one- and two-year period (5). Nevertheless the AUCs of frailty instruments are generally not very convincing (6).

It has been suggested that the combined use of two frailty screening measures could provide complementary information and might increase the predictive power (7, 8). Instruments can be applied sequentially or in parallel. Sequential use means that the second instrument is only applied when the first instrument gives a positive result. When used in parallel, both instruments are applied at the same time. Sequential use maximizes specificity and the positive predictive value, i.e. the probability that a person with positive test results is indeed frail (9). Starting with the test with the highest specificity is most efficient, as it requires fewer persons to undergo both screening measures. In contrast, parallel use maximizes sensitivity and the negative predictive value. By applying the two instruments at the same time, frailty will be less likely to be missed and the results are more rapidly available.

The aim of our study was to investigate whether the combined use of available frailty screening instruments, sequential and parallel, would result in a better prediction of frailty in terms of (I)ADL dependency, mortality and hospitalization compared to the use of a single frailty instrument.

## Methods

We conducted a prospective cohort study with a two-year follow-up. The study was approved by the medical ethical committee of Zuyderland and Zuyd University of Applied Sciences in the Netherlands (METC Z, 12-N-129).

### Participants

A detailed description of the selection of participants is provided elsewhere (10). Briefly, 56,000 people aged 55 years and over, living in the province of Limburg, a southern region of the Netherlands, received first an extensive general health questionnaire sent out by the Dutch Community Health Services. The respondents, who were at least 65 years old and pre-frail or frail, according to Fried's frailty criteria, were then asked to participate in our study. In total, 2,420 persons gave informed consent and participated in the baseline of the present study. Gender, age, living situation and educational level were assessed at baseline.

### Frailty instruments

For the combined use of the two frailty instruments, combinations of four different frailty screening instruments were tested. Instruments with high specificity values (Frailty Phenotype [FP], Frailty Index [FI]), as presented in previous research (4), were combined with instruments with high levels of sensitivity (Tilburg Frailty Indicator [TFI], Groningen Frailty Indicator [GFI]), resulting in four combinations that were investigated: FP-TFI, FP-GFI, FI-TFI and FI-GFI.

The FP, as described by Fried and colleagues, includes five criteria (weight loss, exhaustion, physical activity, walk time and handgrip strength) for the identification of physical frailty among older people (11). Questions about weight loss and exhaustion were asked as proposed by Fried and colleagues. The Short Questionnaire to Assess Health-enhancing physical activity (SQUASH) was used to determine the physical activity criterion (12). Walk time and handgrip strength were measured with the self-report questions ‘Can you reach the other side of the road when the light turns green at a zebra crossing?' and ‘Do you experience difficulties in daily life because of low grip strength?' respectively, rather than using a performance based measure. A detailed description of the self-report measures for these criteria can be found elsewhere (13). Theoretical scores range from 0 to 5 and classify individuals into non-frail (score 0), pre-frail (score 1.2) or frail (score 3.5). As mentioned previously, only pre-frail and frail persons were included in the baseline assessment of the present study.

The FI, developed by Rockwood and Mitnitski, is characterized by a non-fixed set of so-called ‘deficits' (14). We created an FI using the guidelines provided by Searle and colleagues (15). First, we chose all available items from the questionnaire sent by the Dutch Community Health Services, that were presumably related to frailty. We selected 61 potential items that covered several topics, such as (chronic) diseases, loneliness, physical limitations and psychological distress. All items were then dichotomized into the presence ‘1' or absence ‘0' of the item. Next, items with a prevalence of less than five percent were excluded, as proposed by Drubbel and colleagues (16). Finally, we ended up with an FI that consisted of 53 items. The final score of the FI can be calculated by dividing the number of deficits present by the total number of deficits that are measured. Theoretical scores range from 0 to 1, with higher scores indicating a higher level of frailty. A cut-off value of 0.25 was used to distinguish between frail and non-frail individuals (17).

The TFI was developed by Gobbens and colleagues (18). This 15-item questionnaire comprises items in the physical (8 items), psychological (4 items) and social (3 items) domains. Theoretical scores range from 0 to 15, with higher scores indicating a higher level of frailty. A person is considered frail with a score of ≥ 5 (18).

The GFI was developed by Steverink and colleagues (19). This 15-item questionnaire comprises items in the physical (9 items), cognitive (1 item), social (3 items) and psychological (2 items) domains. Theoretical scores range from 0 to 15, with higher scores indicating a higher level of frailty. Persons with a score ≥ 4 are considered frail (20).

### Outcome measures

The outcome measure (I)ADL dependency was defined as an increase in having to depend on someone else when performing (instrumental) activities of daily living, which was determined by the Groningen Activity Restriction Scale (GARS) (21) at baseline and after two years. The GARS is composed of 18 questions about the degree to which someone is able to perform ADL and IADL activities independently. The four response options for each activity are: 1. ‘Yes, I can do it fully independently without any difficulty', 2. ‘Yes, I can do it fully independently but with some difficulty', 3. ‘Yes, I can do it fully independently but with great difficulty' 4. ‘No, I cannot do it fully independently, I can only do it with someone's help'. For each question, the results were dichotomized into being independent (options 1.3) or dependent (option 4), as described in the GARS manual (22). Changes over time per item were then analysed. An increase in dependency was defined as more changes from independent to dependent than vice versa over the two-year observation period.

Data on mortality (deceased yes/no) at two-year follow-up were provided by Statistics Netherlands. The outcome hospitalization was dichotomized into ‘Yes' when someone was admitted at least once to a hospital during the study period, or ‘No' when no hospital admission had taken place.

### Statistical analysis

Missing values were handled as proposed in prior research. Case mean substitution was applied when missing items were less than 25% for the TFI and GFI (23) and 50% for the GARS (21). On the FP, one missing value was allowed when a person had a valid score of 0–2 and two missing values were allowed if the total score was ≥3 (13). For the FI, the non-missing population mean of an item was imputed for each missing item (24).

Descriptive statistics were computed to provide information on the characteristics of the study population. Cut-off values for frailty were used as proposed by the developers of the instruments. Analyses regarding the sequential use of instruments were conducted as follows: first, participants were selected who were frail according to a specific frailty instrument; second, of these frail participants, only those who were also frail based on a sensitive frailty instrument were finally classified as frail. All others were considered nonfrail. For analyses regarding the parallel use of instruments, participants were considered frail when at least one of the two instruments classified them as frail. Participants were only considered non-frail when they were non-frail according to both frailty instruments. Sensitivity, specificity, positive and negative predictive values were then calculated for each single instrument and for the combined instruments (both sequential and parallel), for all three outcome measures.

All analyses were performed using SPSS 22 (IBM SPSS Statistics for Windows, IBM Corp., Armonk, NY).

## Results

In total, 2,420 persons participated in the study. Their mean age was 76.3 ± 6.6 years and 60.5% were females. Additional baseline characteristics are presented in Table 1. At the two-year follow-up, data on changes in (I)ADL dependency were available for 1,872 individuals of whom 35.7% experienced an increase in dependency. Hospitalization was reported by 836 participants (46.4% of 1,803 valid cases) and 182 participants (7.5% of 2,420 valid cases) died during the study period. Missing data for the outcomes (I)ADL dependency and hospitalization were partly due to mortality (n = 182) and admission to a long-term care facility (n = 53). The remaining participants were lost to follow-up for other (unknown) reasons (n = 313 for (I)ADL dependency and n = 382 for hospitalization).

**Table 1:** Baseline characteristics of the study population

		Value
n		2420
Age	mean ± SD	76.3 ± 6.6
Female	n (%)	1463 (60.5)
Living situation		
Living alone	n (%)	906 (39.2)
Not living alone	n (%)	1404 (60.8)
Educational level*		
Low	n (%)	1579 (68.9)
High	n (%)	714 (31.1)
FP		
Score	mean ± SD	1.8 ± 1.0
Frail	%	22.2
FI		
Score	mean ± SD	0.20 ± 0.12
Frail	%	30.2
TFI		
Score	mean ± SD	6.0 ± 3.3
Frail	%	64.8
GFI		
Score	mean ± SD	4.6 ± 3.0
Frail	%	59.3
Dependent on at least 1 GARS item	n (%)	1472 (61)


FP: Frailty Phenotype; FI: Frailty Index; TFI: Tilburg Frailty Indicator; GFI: Groningen Frailty Indicator; GARS: Groningen Activity Restriction Scale; SD: Standard deviation; * Low educational level = no education, completion of primary school or pre-vocational secondary education; high educational level = higher than primary school or prevocational secondary education

**Figure 1:**
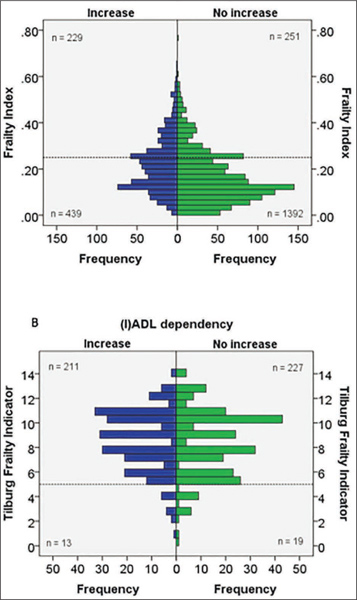
Sequential use of the Frailty Index (FI) and the Tilburg Frailty Indicator (TFI) for the outcome increase in dependency in (instrumental) activities of daily living ((I)ADL). A) Distribution of all participants who did and did not experience an increase in (I)ADL dependency on the FI. B) Distribution of individuals, who were frail on the FI, who did or did not experience an increase in (I)ADL dependency on the TFI. Cut-off values are presented as dotted lines

The sequential use of two frailty instruments is presented in Figure 1. Graph A displays the distribution of all participants (n = 1,872) who did and did not experience an increase in (I) ADL dependency on the FI, the specific instrument. Only those classified as frail (n = 480) are included in graph B, which shows the distribution of persons who did and did not experience an increase in (I)ADL dependency on the TFI, the sensitive instrument. Similar results were found for the other sequential combinations of frailty instruments.

Sensitivity, specificity, positive predictive value (PPV) and negative predictive value (NPV) for the single and the combined instruments for (I)ADL dependency are presented in Table 2. For the single instruments, the FP and FI showed higher values of specificity, whereas the TFI and GFI had higher values of sensitivity. As expected, the sequential use of two frailty instruments resulted in lower levels of sensitivity and NPV, together with higher levels of specificity and PPV. However, the degree of change for the PPV and NPV was slight. The parallel use of the two frailty instruments, in general,resulted in high levels of sensitivity and NPV, together with lower levels of specificity and PPV. The PPV and NPV again changed only slightly, as in the other combination. Comparable results were found for the outcomes hospitalization and mortality (see online Supplement 1).

**Table 2:** The number of frail persons at baseline and sensitivity, specificity, positive predictive value (PPV) and negative predictive value (NPV) of the four single frailty instruments and the combined frailty instruments (sequential and parallel) for the outcome (I) ADL dependency at two-year follow-up

	Frail according to instruments (n, baseline)	Sensitivity (%)	Specificity (%)	PPV (%)	NPV (%)
*Single instrument*					
FP	537	24.7	86.2	49.8	67.4
FI	730	34.3	79.2	47.7	68.5
TFI	1536	72.7	45.7	42.6	75.2
GFI	1424	66.0	51.1	42.8	73.1
*Sequential*					
FP & TFI	485	23.1	87.9	51.3	67.4
FP & GFI	464	21.6	88.5	51.1	67.0
FI & TFI	663	31.8	81.1	48.2	68.3
FI & GFI	651	30.6	81.4	47.8	67.9
*Parallel*					
FP &TFI	1567	73.8	44.4	42.3	75.4
FP & GFI	1490	69.0	49.0	42.8	74.1
FI & TFI	1580	74.7	44.1	42.5	75.9
FI & GFI	1495	69.6	49.0	43.0	74.5


FP: Frailty Phenotype; FI: Frailty Index; TFI: Tilburg Frailty Indicator; GFI: Groningen Frailty Indicator

## Discussion

The aim of our study was to investigate whether the combined use of frailty instruments, either sequential or parallel, would result in a better prediction of (I)ADL dependency, mortality and hospitalization, compared to the use of a single frailty instrument. In our study, we were unable to demonstrate a clear beneficial effect of using either combination of frailty instruments. As expected, specificity levels increased when applying the instruments sequentially; however, the PPV hardly increased. The parallel use of two instruments increased sensitivity; however, the NPV hardly increased.

To the best of our knowledge, this is the first study to investigate the possible value of the combined application of two frequently used frailty screening instruments. In some other studies, a frailty instrument has been combined with another measurement. For instance, Kenig and colleagues examined frailty (defined by deficits in two or more domains of the comprehensive geriatric assessment) and the Surgical Apgar Score (25). Compared to the individual instruments, the combination did not increase the PPV for 30-day morbidity and only slightly increased the NPV for 30-day mortality among older patients undergoing abdominal cancer surgery. Also, frailty screening can be followed by a more thorough assessment. For example, the ‘Prevention of Care' programme comprises screening with the GFI (26). When someone scores 5 or higher, a multidimensional assessment is conducted by a practice nurse at the patient's home to gain insight into problems in performing daily activities and risk factors for disability. However, the screening instruments used in such approaches often include many false-positive cases, which render them inefficient, and the second steps are often very time consuming. In these cases, the sequential use of two screening instruments might be relevant.

A major strength of this study is the simultaneous assessment of four available frailty instruments in a large cohort of community-dwelling older people, which is the best strategy for comparing the performance of instruments. In particular, PPV and NPV, which are affected by the prevalence of the outcomes, are difficult to compare when the results are obtained from different studies. By applying instruments sequentially, a higher PPV can be achieved (9). At the same time, it also causes more false-negative cases, indicating that frail persons are missed in screening. One might utilize this strategy, for example, when costly or time-consuming clinical management follows in terms of advanced diagnostics or expensive treatment. On the other hand, while parallel testing increases the NPV, it causes more false-positive cases. This method would be best applied if one desired to include as many frail persons as possible, for research purposes or in daily practice. However, follow-up and interventions would then often be applied to those not needing extensive monitoring.

Our study population consisted of pre-frail and frail patients and did not include non-frail persons. In daily practice, frailty instruments are most often applied by healthcare professionals in persons who are at risk of becoming frail. The inclusion of pre-frail and frail persons makes our population more reflective of the persons for whom frailty measures are useful rather than persons sampled from the general population. Nevertheless, for the selection of the cohort the FP was used, which focusses on the physical aspects of frailty. Persons that were frail in other domains (e.g. psychological or social) might therefore have been excluded, which may have influenced the results.

All frailty instruments were assessed as proposed by the developers, except for the FP, for which we used self-report questions instead of performance-based measures, potentially having a slight influence on the results (27). In our study, the FP and FI were handled as specific instruments and the TFI and GFI as sensitive instruments (4). Some studies, however, show other values of sensitivity and/or specificity (5, 28). The combined use of instruments should therefore be studied further with different instruments (with high levels of sensitivity and/or specificity), in other study populations and/or with different (handling of) outcome measures. One of the instruments that might be interesting to investigate is the Vulnerable Elders Survey (VES)-13 (29). In a recent study of Bongue and colleagues this instrument demonstrated very high levels of sensitivity for various outcome measures (30). Moreover, this instrument has often been cited over the past years and is thus of interest to many researchers (31). Regarding the investigation of another study population, the oldest old (80+ years) could be considered. Frailty is more present among people in this age group and older people are more at risk for adverse health outcomes compared to younger ones. An example of a different handling of an outcome measure is the number of hospital admissions. From the participants who reported to be admitted to a hospital in our study, 355 (42%) were admitted once, 196 (23%) twice, and 227 (27%) three times or more (missing values: n = 58 (7%)). Clearly there is a large variation in the number of admissions. Hospital admissions can be caused by factors unrelated to frailty. It is unknown if multiple admissions are more often related to frailty compared to one admission and if combined use of frailty instruments can predict multiple admissions.

Based on our results, we conclude that the combined application of two frailty instruments might not be a solution to achieve a better identification of frailty in community-dwelling older people. However, as this is one of the first studies to investigate the combined use of screening instruments, we recommend further exploration of other combinations of instruments in various study populations.

*Funding:* This work was supported by the Nationaal Regieorgaan Praktijkgericht Onderzoek SIA (project number PRO -1-007) and Zuyd University of Applied Sciences. Both organizations had no role in the design and conduct of the study; in the collection, analysis, and interpretation of data; in the preparation of the manuscript; or in the review or approval of the manuscript.

*Conflict of interest:* Drs. Op het Veld has nothing to disclose. Dr. van Rossum has nothing to disclose. Dr. Kempen has nothing to disclose. Dr. Beurskens has nothing to disclose. Dr. Hajema has nothing to disclose. Dr. de Vet has nothing to disclose.
