# Supplementary material for: Can the Combined Use of Two Screening Instruments Improve the Predictive Power of Dependency in (Instrumental) Activities of Daily Living, Mortality and Hospitalization in Old Age?
Source: J Frailty Aging. 2019 Jun 12;8(4):180–5. doi: 10.14283/jfa.2019.17 (PMC12275724; doi:10.14283/jfa.2019.17)
Supplement: Supplementary file 1 — Supplement 1 [file mmc1.pdf]

## Supplement 1

The number of frail persons at baseline and sensitivity, specificity, positive predictive value (PPV) and negative predictive value (NPV) of the four single frailty instruments and the combined frailty instruments (sequential and parallel) for the outcomes mortality and hospitalization at two-year follow-up.

**Mortality**

| | Frail according to instruments (n, baseline) | Sensitivity (%) | Specificity (%) | PPV (%) | NPV (%) |
|---|---|---|---|---|---|
| *Single instrument* | | | | | |
| FP | 537 | 44.5 | 79.6 | 15.1 | 94.6 |
| FI | 730 | 49.5 | 71.4 | 12.3 | 94.6 |
| TFI | 1536 | 80.6 | 36.5 | 9.4 | 95.8 |
| GFI | 1424 | 76.2 | 42.1 | 9.7 | 95.6 |
| *Sequential* | | | | | |
| FP &TFI | 485 | 42.8 | 81.6 | 15.9 | 94.6 |
| FP & GFI | 464 | 39.8 | 82.4 | 15.5 | 94.4 |
| FI & TFI | 663 | 46.7 | 73.9 | 12.8 | 94.4 |
| FI & GFI | 651 | 46.2 | 74.6 | 12.9 | 94.4 |
| *Parallel* | | | | | |
| FP &TFI | 1567 | 81.7 | 35.2 | 9.4 | 95.9 |
| FP & GFI | 1490 | 80.7 | 39.5 | 9.8 | 96.2 |
| FI & TFI | 1580 | 83.3 | 34.7 | 9.5 | 96.2 |
| FI & GFI | 1495 | 79.6 | 39.1 | 9.6 | 95.9 |

**Hospitalization**

| | Frail according to instruments (n, baseline) | Sensitivity (%) | Specificity (%) | PPV (%) | NPV (%) |
|---|---|---|---|---|---|
| *Single instrument* | | | | | |
| FP | 537 | 25.6 | 86.2 | 61.7 | 57.3 |
| FI | 730 | 35.9 | 79.6 | 60.4 | 59.0 |
| TFI | 1536 | 70.5 | 44.1 | 51.8 | 63.7 |
| GFI | 1424 | 63.9 | 50.3 | 52.6 | 61.7 |
| *Sequential* | | | | | |
| FP &TFI | 485 | 23.5 | 87.6 | 61.9 | 57.1 |
| FP & GFI | 464 | 23.1 | 88.6 | 63.7 | 57.1 |
| FI & TFI | 663 | 32.9 | 81.2 | 60.1 | 58.5 |
| FI & GFI | 651 | 32.7 | 82.2 | 61.3 | 58.5 |
| *Parallel* | | | | | |
| FP &TFI | 1567 | 72.0 | 43.1 | 51.9 | 64.3 |
| FP & GFI | 1490 | 66.3 | 48.1 | 52.4 | 62.3 |
| FI & TFI | 1580 | 73.0 | 42.8 | 52.1 | 65.0 |
| FI & GFI | 1495 | 67.1 | 48.0 | 52.7 | 62.8 |

**FP= Frailty Phenotype, FI= Frailty Index, TFI= Tilburg Frailty Indicator, GFI= Groningen Frailty Indicator**
